# Minimally invasive surgery: a historical and legal perspective on technological transformation

**DOI:** 10.1007/s11701-025-02589-7

**Published:** 2025-07-21

**Authors:** J. Ravichandran Jeganathan, Ravindran Jegasothy, Woon Teen Sia

**Affiliations:** 1 Monash Medical University, Petaling Jaya, Malaysia; 2 Hospital Sultanah Aminah, Johor Bahru, Johor Malaysia; 3 MAHSA University, Jenjarom, Malaysia

**Keywords:** Minimally invasive surgery (MIS), Robotic surgery, Artificial intelligence in surgery, Medicolegal issues, Surgical innovation and ethics

## Abstract

Minimally Invasive Surgery (MIS) has experienced a significant evolution over the last 5,000 years, progressing from basic manual methods to sophisticated, robot-assisted approaches. The evolution of minimally invasive surgery (MIS) has been influenced by significant advancements in endoscopic visualization, electrosurgery, and laparoscopic tools, while recent innovations in artificial intelligence (AI) and robotic systems have further augmented surgical accuracy, minimized operative trauma, and enhanced patient outcomes. Notwithstanding its therapeutic advantages, minimally invasive surgery presents considerable medicolegal complications. The escalating intricacy of surgical technologies and procedures has resulted in an increased possibility of malpractice claims, presenting significant financial and professional hazards to healthcare providers. This requires the establishment of effective risk mitigation techniques, encompassing thorough surgical training, credentialing procedures, and compliance with established clinical standards. This review is divided into three sections: (1) the historical development and technological milestones of MIS; (2) the present landscape and future trajectories, emphasizing AI and robotic integration; and (3) the ethical and legal implications associated with MIS advancements, encompassing informed consent, surgeon liability, and patient safety concerns. The document underscores the pressing necessity for the advancement of legal and ethical frameworks to align with technological progress. Balancing innovation with ethical and legal safeguards is essential to ensure both progress and protection in modern surgical practice.

## Introduction

Minimally Invasive Surgery (MIS) represents one of the most significant paradigmatic shifts in the surgical history. Based on the National Center for Biotechnology Information (NCBI), MIS is defined as a procedure with minimal damage to the entry point (e.g., skin, body cavity, and anatomical opening) [1]. Current literature highlights the association of MIS with less pain, lower post-operative morbidity, and faster recovery than the conventional approach to the same operation [2]. The emergence and evolution of minimally invasive surgery (MIS) is the true portrayal of a dynamic and continuous process in the advancement of healthcare technology and technique, leading to changes in practice [3]. The innovations of MIS include instrumental modifications (e.g., bipolar Maryland dissector, short needle holder, and endoloop ligature) and MIS-associated technological development (e.g., robotics, Single-Incision Laparoscopic Surgery SILS, Natural Orifice Transluminal Endoscopic Surgery NOTES, and Artificial Intelligence AI integration).

However, alongside its clinical benefits, MIS has introduced a complex set of medicolegal and ethical challenges. Increased technical complexity, steep learning curves, and evolving standards of care contribute to potential errors and litigation risks. Additionally, the rapid pace of innovation often outpaces regulatory and training frameworks, leaving gaps in surgeon credentialing, informed consent, and institutional oversight. These issues are particularly critical as the medical community faces rising litigation costs, patient safety demands, and the growing influence of artificial intelligence (AI) in surgical decision-making.

This narrative review aims to provide a comprehensive overview of the evolution of MIS from its historical origins to its modern legal and ethical landscape. We highlight major milestones in MIS development, examine current medicolegal vulnerabilities with a focus on gynecological surgery, and explore future trends including AI and robotic integration. By combining historical insight with contemporary legal analysis, this review offers a timely reflection on the need for balanced surgical innovation—one that is technologically progressive yet ethically grounded and legally accountable.

## Methodology

This article is a narrative review synthesizing the historical evolution, contemporary practice, and medicolegal considerations of minimally invasive surgery (MIS). Given the multidisciplinary scope—spanning history, surgical innovation, medical law, and ethics—a narrative approach is chosen to provide flexibility in integrating diverse sources and perspectives.

A comprehensive literature search was conducted using electronic databases, including PubMed, Google Scholar, SpringerLink, Scopus, and ScienceDirect. Search terms included “minimally invasive surgery,” “laparoscopy,” “robotic surgery,” “surgical history,” “medical ethics,” “informed consent,” “surgical negligence,” “Bolam test,” “Montgomery ruling,” “AI in surgery,” and related terms. Both historical and contemporary sources are considered, including peer-reviewed journal articles, surgical textbooks, legal case law summaries, and position statements from professional bodies (e.g., SAGES, American College of Surgeons).

The time frame for literature selection ranged from ancient surgical practices (~ 3000 BC) to contemporary publications up to 2024. Articles and documents are included if they [1] contributed substantially to the understanding of MIS development [2], addressed legal or ethical issues in MIS, or [3] presented relevant technological milestones. Legal cases and guidelines from jurisdictions, such as the UK, US, and Malaysia, were included for comparative insights. Non-English articles, duplicate reports, and irrelevant commentaries were excluded.

### Part 1: evolution of minimally invasive surgery

#### Exploring the body cavities: the dawn of endoscopy

While MIS is often regarded as a hallmark of modern medicine, its roots can be traced back to approximately 5000 years ago, with early civilizations developing instruments to examine and treat internal structures [4]. The Sumerians (3000 BC) used copper knives and gold malleable catheters for surgery, while the Egyptians (2400 BC) crafted bronze medical instruments (e.g., scalpels, needles) [4, 5]. Babylonians and Assyrians (1300 BC) employed hollow copper tubes for medication delivery, with the early use of speculum mentioned in the Babylonian Talmud [4, 6]. In India (800–600 BC), Sushruta, the Father of Surgery, designed endoscopes and rectal specula with openings at either or both ends [7, 8]. Greek and Roman physicians refined these instruments, with Hippocrates and his colleague using catheters and rectal specula, Erasistratus and Oreibasis advancing catheter designs and Archigenes introducing cervical mirrors [5, 9, 10]. Arab surgeon Al Zahrawi (936–1009 AD) later developed a screw-operated glass speculum, a mechanism still found in modern devices [11]. The first use of an artificial light source was introduced by Guilio Cesare Aranzi in 1587 [12]. In the seventeenth and eighteenth centuries, medical innovators, such as George Arnaud de Ronsil and Philipp Bozzini, played a crucial role in improving early endoscopic light sources [13]. They developed light-focusing devices that enabled the examination of deeper body cavities, enhancing diagnostic precision. In particular, Bozzini, created Der Lichtleiter (light conductor) in 1806, a groundbreaking instrument that used candlelight and mirrors to illuminate internal structures [4]. In the early nineteenth century, French surgeon Jean Civiale revolutionized urology by developing lithotripsy. [14]. Later, Antonin Jean Desormeaux improved endoscopic visualization with a portable cystoscope with an enhanced light source, earning him the title “Father of Endoscopy” [15, 16].

**Fig. 1:**
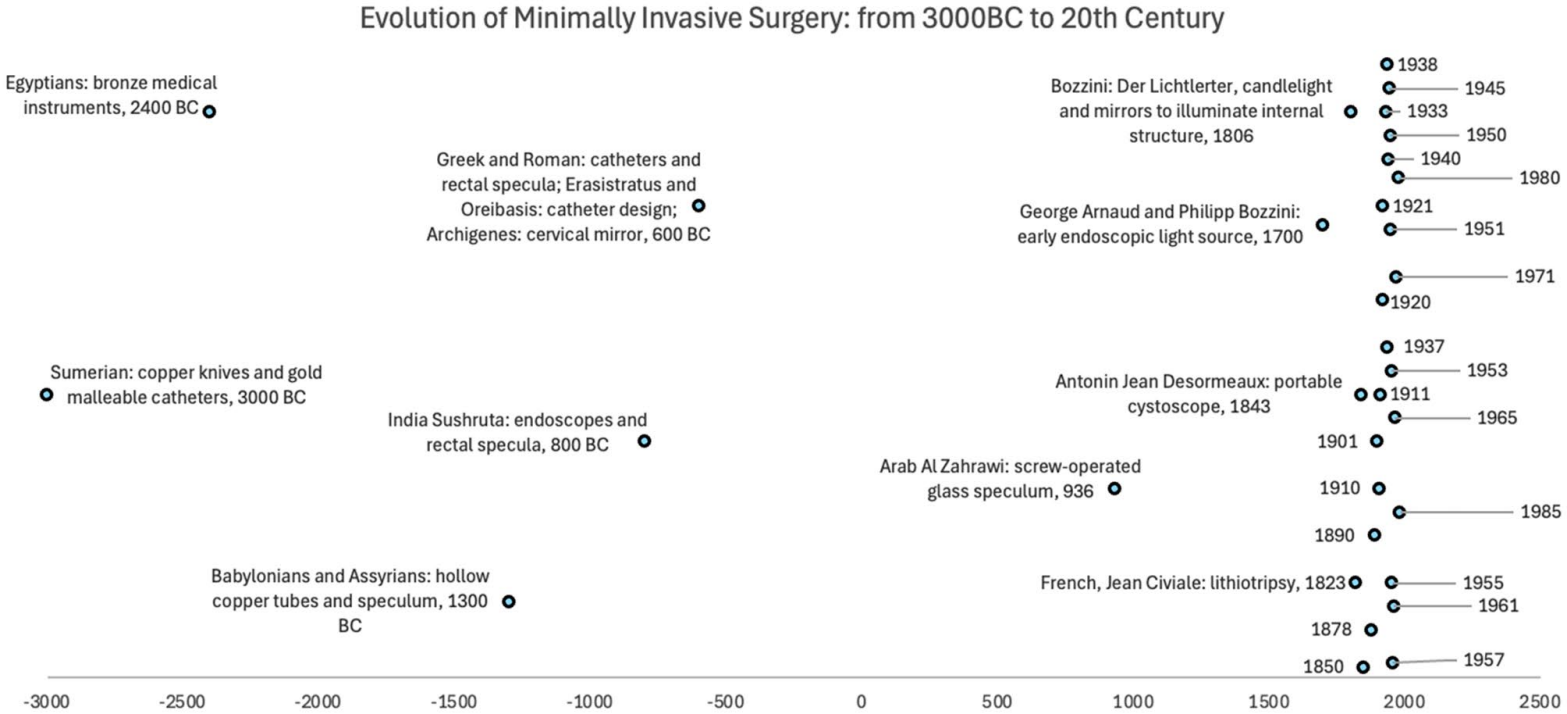
Evolution of MIS: from 3000BC to twentieth century

In brief, from 3000 BC to 1850, the early medical tools evolved to enhance the visualization of the body cavities, paving the way for minimally invasive techniques. These ancient innovations represent more than historical curiosity; they illustrate enduring priorities in MIS, such as access, visibility, and patient tolerance. Even in antiquity, procedures required balancing diagnostic benefit with procedural risk, foreshadowing today’s ethical concerns around consent, and procedural safety in MIS (Fig. 1).

#### Innovations unfold: from candlelight to cutting-edge technology

**Fig. 2:**
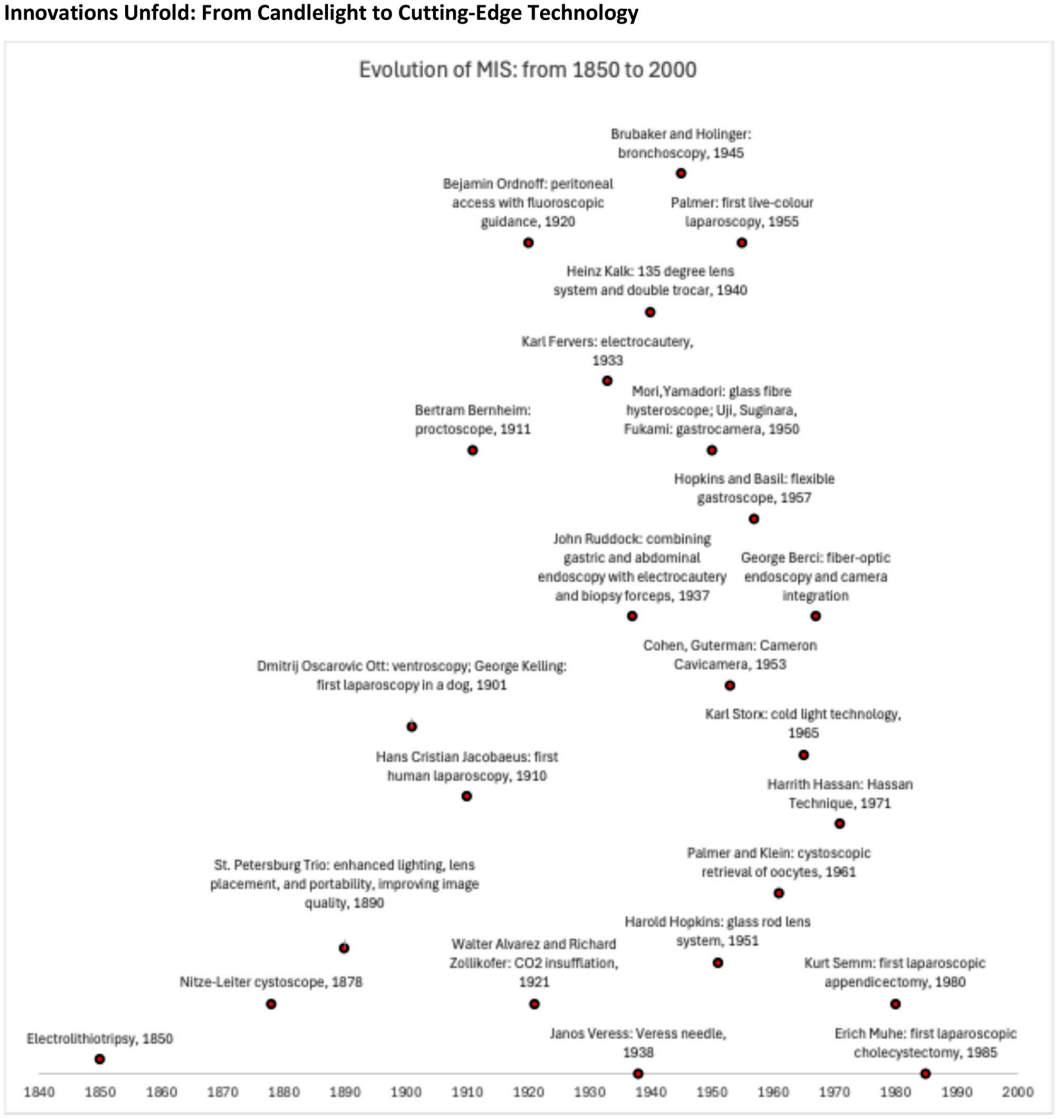
Evolution of MIS: from 1850 to 2000

In the mid-nineteenth century, electricity was introduced into surgery with electrolithotripsy. George Robinson used a Leyden jar to break bladder stones, while Franz von Paula Gruithuisen had similarly experimented with insulated platinum wires for a similar purpose. [4]. In the late nineteenth century, Maximilian Nitze revolutionized endoscopy with the Nitze-Leiter cystoscope (1878), featuring an electrically heated platinum wire for illumination, marking the shift from candlelight to electric light following Edison’s invention of the light bulb (patented in 1879). The St. Petersburg Trio—Alexander Ebermann, Alfred Couriard, and Benjamin Tarnowsky—enhanced lighting, lens placement, and portability, improving image quality [17]. Dmitrij Oscarovic Ott further advanced minimally invasive surgery by pioneering ventroscopy [18]. Advancements of laparoscopy in the inspection of larger body cavities (i.e., abdomen, thorax) emerged in the early twentieth century. Georg Kelling (1901) performed the first laparoscopy in a dog using a modified cystoscope and air insufflation (coelioscopy) [19]. Soon after, Hans Christian Jacobaeus (1910) advanced the technique by conducting the first human laparoscopy and thoracoscopy for diagnostic purposes [19]. In 1911, Bertram Bernheim introduced laparoscopy in the USA using a proctoscope, marking a significant step toward minimally invasive inspection of larger body cavities (Fig. 2).

Significantly, gynecologists led the field primarily, driving advancements in organ visualization, laparoscopic indications, pneumoperitoneum techniques, electrocautery, and optical systems. Walter Alvarez (1921) and Richard Zollikofer (1924) introduced CO₂ insufflation, reducing pain and explosion risks from air/oxygen use [20]. Janos Veress (1938) developed the Veress needle for safer cavity access, while Benjamin Ordnoff (1920) pioneered peritoneal access under local anesthesia with fluoroscopic guidance [21, 22]. In optics and instrumentation, Heinz Kalk (1940s) enhanced visualization with a 135° lens system and double trocar, improving liver and gallbladder examination [23]. Karl Fervers (1933) introduced electrocautery for therapeutic laparoscopy, though early use posed explosion risks with oxygen insufflation. Harrith Hasson (1971) revolutionized laparoscopic access with open laparoscopy, emphasizing direct visualization to minimize injury risks, now a standard technique [24]. In addition to the surgical techniques, the integration of cinematic and television technology played a crucial role in the advancement of laparoscopy. Japanese innovators Mori and Yamadori introduced glass-fiber hysteroscopes for live childbirth imaging [20]. In 1950, Uji, Suginara, and Fukami’s development of the gastrocamera revolutionized gastrointestinal endoscopy. Around the same time, Cohen and Guterman (1953) introduced the Cameron cavicamera for surgical imaging, enhancing both still and moving-picture documentation [20]. The educational potential of laparoscopy was further demonstrated by Palmer’s first live-color laparoscopy in 1955, following earlier bronchoscopy films by Brubaker and Holinger in 1945 [20].

These nineteenth-century innovations demonstrate a critical shift—from merely entering body cavities to actually visualizing them safely and consistently. The introduction of artificial lighting significantly enhanced procedural control, reducing the reliance on tactile or blind techniques. However, these advancements also introduced new dependencies on evolving technology—an issue that still underlies many claims of equipment-related MIS complications today.

Gynecological laparoscopy also experienced significant progress, particularly in the refinement of sterilization techniques and the introduction of carbon dioxide for insufflation. Innovations in lighting systems and structured training programs greatly impacted reproductive surgery. In 1961, Palmer and Klein successfully retrieved an oocyte laparoscopically using a cystoscope, a breakthrough in assisted reproduction [25]. This period also marked the refinement of tubal ligation using monopolar and later bipolar diathermy, with 25 mmHg established as the maximum safe intra-abdominal pressure. Advancements in minimally invasive surgery continued into the late twentieth century. In 1980, Kurt Semm performed the first laparoscopic appendectomy, a milestone that reshaped general surgery [26]. Five years later, Erich Mühe pioneered laparoscopic cholecystectomy using his "gallscope," initially facing scepticism but later recognized as the foundation of modern gallbladder surgery. Alongside these procedural advancements, fiber-optic and imaging technology significantly improved surgical precision. Harold Hopkins’ glass rod lens system, developed in 1951, enhanced image clarity and was later integrated with Karl Storz’s cold light technology in 1965, forming the backbone of contemporary endoscopy [27, 28]. Hopkins also collaborated with Basil Hirschowitz to develop the first flexible gastroscope in 1957, expanding diagnostic capabilities [29]. In parallel with these innovations, Hungarian–American surgeon George Berci worked closely with Karl Storz and helped develop the first video-laparoscopy system, enabling real-time visualization. His impact was recognized by the American College of Surgeons with the Jacobson Innovation Award in 2011, underscoring his role as a central figure in the evolution of MIS.

Beyond imaging advancements, John Ruddock contributed to the field by combining gastric and abdominal endoscopy with electrocautery and biopsy forceps, bridging the gap between diagnostic and interventional endoscopy [30, 31]. Meanwhile, Benjamin Ordnoff introduced radiologically guided laparoscopy, prioritizing safety and diagnostic accuracy [22]. The evolution of video-assisted surgery further expanded laparoscopic applications, allowing real-time projection of procedures onto monitors. This facilitated the refinement of laparoscopic suturing techniques and enabled the execution of complex procedures, such as bowel resections and radical hysterectomies, solidifying laparoscopy as a cornerstone of modern surgery.

Innovations like CO2 insufflation and the Veress needle lay the structural and physiological foundations for modern MIS. Today’s medicolegal scrutiny of insufflation injuries and access-related complications echo the clinical challenges in this era. Standardization of the access techniques is currently a major point of credentialing and surgical training. In addition, the significantly improved image clarity and depth perception which directly address the early limitations of surgical visualization have improved operative safety. However, the dependence on evolving visual technologies also mean that insufficient training or equipment failure could compromise outcomes, as seen in litigation involving optical misidentification or missed intraoperative diagnoses. The landmark procedures like first laparoscopic appendicectomy and cholecystectomy usher MIS into mainstream general surgery. Their initial reception, marked by scepticism, litigation risk, and institutional resistance, mirrors the current hesitation around newer platforms such as Natural Orifice Transluminal Endoscopic Surgery (NOTES) and AL-assisted surgeries.

#### Beyond the surgeon’s hands: organizational challenges and the rise of the robots

The 1990s marked a turning point in minimally invasive surgery with the formation of professional organizations such as the Society of Laparoscopic Surgery (SLS) and the European Association of Endoscopic Surgery (EAES) [32]. In the United States, the Society of American Gastrointestinal and Endoscopic Surgeons (SAGES), founded in 1981, played a pivotal role in standardizing laparoscopic techniques and developing educational programs such as the Fundamentals of Laparoscopic Surgery (FLS). Regionally, the Endoscopic and Laparoscopic Surgeons of Asia (ELSA), established in 1990, promoted MIS practices across Asia through conferences, training, and cross-border collaboration. On a global scale, the International Federation of Societies of Endoscopic Surgeons (IFSES), formed in 1992, united over ten member societies and acted as an umbrella organization to facilitate global dialog, innovation dissemination, and harmonization of surgical standards. These societies continue to shape the field by advocating for safety, ethics, and the responsible integration of new technologies. They foster collaboration, mentorship, and the standardization of techniques.

However, the rapid adoption of laparoscopic surgery brought challenges, including iatrogenic injuries, prompting the development of quality assurance measures, safety protocols, and structured training programs. Initiatives like LAPCO (i.e., a national training program of laparoscopic colorectal surgery in England) helped standardize laparoscopic colorectal surgery, addressing ongoing debates over techniques and outcomes. Clinical trials, such as the CLASSIC and COLOR studies, further validated the efficacy of laparoscopic approaches in colorectal cancer, demonstrating comparable survival rates to open surgery while offering additional patient benefits.

Collaboration between surgeons and industry has also played a pivotal role in advancing technology, with companies like Storz leading innovations in surgical instrumentation [33]. The advent of robotic surgery, particularly with the approval of the Da Vinci system, has further transformed the field, enhancing precision and ergonomics. While robotics have proven beneficial in procedures like prostatectomy, their application has expanded significantly into general surgery, including colorectal resections, hiatal and inguinal hernia repairs, cholecystectomies, and bariatric surgeries. These systems offer enhanced three-dimensional visualization, improved ergonomics, tremor filtration, and increased precision in confined anatomical spaces. However, its widespread application in surgery remains controversial due to high costs and inconsistent clinical outcomes [34].

### Part 2: MIS today and advancing into the future

MIS has become a cornerstone of modern surgical practice, with widespread adoption across multiple specialties, including general, gynecologic, urologic, and oncologic surgery. As training programs increasingly mandate proficiency in these techniques, laparoscopic surgery continues to evolve, particularly in colorectal procedures. The UK’s National Bowel Cancer Audit has reported a significant rise in laparoscopic colorectal resections, from approximately 25% in 2008–2009 to 61% in 2017–2018, reflecting the growing preference for minimally invasive approaches [32, 35].

Despite its advantages, MIS faces ongoing challenges with emerging techniques, such as Single-Incision Laparoscopic Surgery (SILS) and Natural Orifice Transluminal Endoscopic Surgery (NOTES). While SILS offers cosmetic benefits and fewer port-site complications, its limited maneuverability often results in longer operative times [36]. NOTES, which eliminates external scars by accessing the surgical field through natural orifices like the mouth, rectum, or vagina, remains limited by technical difficulties and sepsis risks [37]. Robotic surgery has also gained traction, particularly with the Da Vinci system, which is now standard for prostate cancer surgery and increasingly used for rectal cancer and bariatric procedures. While robotics enhances precision and ergonomics, its widespread implementation depends on cost reductions, technological advancements, and further clinical validation.

Although early surgical techniques are rudimentary by modern standards, the fundamental challenges they sought to address (e.g., limited access, poor visualization, patient perception) remain central in today’s MIS practice. Ancient tools like specula, early catheters, and illuminated scopes reflect a long-standing drive toward less-invasive intervention. This same goal underpins the modern innovations in laparoscopic and robotic surgeries. Moreover, the ethical considerations such as operator dependency procedural risk and misdiagnosis remain paramount, requiring surgeons to balance technological enthusiasm with patient safety and responsible practice. MIS is not a sudden revolution but the continuum of the culmination of centuries of incremental progress and persistent dilemmas. As advancements continue, the future of MIS will likely see further refinements, making these techniques more accessible, efficient, and ethically sound.

#### Embrace and harness the power of artificial intelligence (AI) in MIS: to own the future of surgery

As the field of MIS continues to evolve, artificial intelligence (AI) is poised to play a transformative role in shaping its future. The integration of AI in robotic-assisted surgery enhances precision through image recognition, motion scaling, and real-time decision support [38]. Al-powered systems like Da Vinci Surgical System is increasingly complemented by machine learning algorithms that assist in anatomical identification and movement optimization, further improves the consistency, efficiency, and overall surgical outcomes [39].

AI-powered machine learning algorithms analyze vast amounts of surgical data, enabling pattern recognition, outcome prediction, and data-driven decision-making to support operating surgeons [40]. Additionally, AI-driven task automation, including suturing and tissue dissection, enhances procedural consistency while reducing the surgeon workload [41]. In laparoscopic procedures, AI has shown promise in tasks, such as automated suturing, real-time risk prediction, and intraoperative guidance, particularly in high-stakes surgeries like colorectal resections. Machine vision, combined with deep learning, enables AI to distinguish tissue planes, reducing iatrogenic injuries and potentially enhancing patient safety. Platforms like Touch Surgery™, The Ethicon AI-powered Laparoscopic Skills Training Platform, and Surgical AI Trainer are actively working toward integrating AI for skill assessment and outcome prediction [42, 43].

Beyond intraoperative applications, AI contributes to the objective evaluation of surgical proficiency by minimizing potential biases associated with human supervision. This capability holds significant value in postgraduate surgical training programs, providing an unbiased and evidence-based approach to skill assessment and competency development.

However, challenges remain. Algorithm transparency, legal responsibility, training data bias, and clinical validation are ongoing concerns. Moreover, AI implementation demands substantial computational infrastructure and raises ethical questions around surgeon autonomy, data ownership, and liability in the event of adverse outcomes. These unresolved medicolegal and practical issues highlight the need for regulatory frameworks that evolve in tandem with technological capability.

Ultimately, while AI is poised to transform MIS, it should be viewed not as a replacement for surgical judgment, but as an adjunct that must be rigorously evaluated, ethically implemented, and continually refined through evidence-based practice.

### Part 3: ethical considerations in MIS

#### Ethical concerns of implementing new technologies and techniques in surgery

The rise of MIS and its expanding roles in the surgical fields have been observed over the past couple decades. Significantly, MIS has become the standard of care for many procedures today (e.g., cholecystectomy, appendectomy, and hernia repair) [44]. A dramatic 462% increase in laparoscopic cases performed by general surgery residents over 18-year period (2000–2018) was reported by Bingmer et al., reflecting the prominence of MIS in the fields [45]. However, the ethical considerations regarding the implementation of new technologies and techniques in surgery remain critical, emphasizing the importance of prioritizing patient safety over technological or financial interests.

Safety is one of the key ethical considerations. The safety of new medical technologies and techniques in the United States (US) is tightly regulated by the Food and Drug Administration (FDA). Over the years, the approval process has become increasingly stringent, resulting in longer approval times, higher costs, and uncertainty. Consequently, start-ups face difficulties securing funding, while many large corporations are discouraged from bringing their innovations into the US market. Alternatively, they choose to market their devices outside the US, where the regulatory processes are perceived as more stable and cost-effective. Eventually, those products may either attempt to obtain US FDA approval or be marketed exclusively outside the US [46]. Unlike medical diagnostics, drugs, and devices, new surgical procedures are not regulated by the US FDA. While federal regulations protect human research subjects, they do not specifically govern innovative procedures. To date, the recommendations to include significant innovations within research projects for patient safety and protection were never formalized into regulations [47]. Instead, the evaluation of new techniques is managed at the institutional level.

The timing and process of implementing MIS are challenging. As there is no universal FDA guidelines, the process of implementing new procedures varies. The balance between the time spent collecting data to support the use of new technologies and the deprivation of care toward patients due to the delay of implementation should be justified [48]. Professional medical associations such as the Society of American Gastrointestinal and Endoscopic Surgeons (SAGES) have published guidelines and executed through committees like the Technology Assessment and Value Assessment Committee (TAVAC) [49]. At the institutional level, the introduction of new procedures is overseen by the local leadership, including department chairs, medical staff, and hospital executives. Many institutions use a multidisciplinary New Technology Committee (NTC) to evaluate new devices and procedures, establish credentialing standards, and review early outcomes. While clinical research is overseen by institutional Review Boards (IRDs), the safe and appropriate integration of new technologies are assessed by NTC. In addition, NTC may assist with patient safety coordination, conflict of interest management, and institutional resource evaluation [47].

Patient autonomy is one of the four pillars of medical ethics. Informed consent is, however, the fundamental requirement that facilitates patients’ self-governance [50]. It can be challenging due to patients’ varying educational and cultural backgrounds, and misinformation from the industry, media, and Internet. While prioritizing patient welfare, surgeons must provide a balanced and transparent discussion about the potential benefits and risks. In the US, efforts are made to standardize the informed consent processes via guidelines from Professional Medical Association (PMA) and NTC, safeguarding surgeons and institutions while ensuring patient protection. A valid informed consent must involve a dialog of understanding, education, and trust-building between surgeons and patients, whereby shared decision-making is made based on values and free of coercion [51, 52].

Adequate training and credentialing for residents and practicing surgeons is another focus to ensure safe implementation of new techniques. Duty-hour restrictions have been imposed in the healthcare system to protect patient safety by mitigating resident fatigue and burnout, promoting resident well-being and ensure adequate training opportunities by providing a structured schedule for rest and learning [53, 54]. However, this might not be the ultimate solution to improving clinicians’ fatigue at work while negatively impacting their clinical and operative experiences, with many residents graduating without sufficient exposure to the core procedures. To address these gaps, simulation-based training and competency-based assessment are becoming more prevalent. The shift from training duration and case numbers to objective competence evaluation is exemplified by the Fundamentals of Laparoscopic Surgery (FLS) program, co-developed by SAGES and the American College of Surgeons (ACS). FLS is now mandatory for US surgical residents and is increasingly used internationally. Some institutions also require FLS certification for laparoscopic surgery privileges, with insurance providers offering incentives for certified surgeons [55]. SAGES is also leading training and credentialing efforts in other areas, including Fundamentals of Endoscopic Surgery (FES), Fundamentals for Use of Energy (FUSE), and Fundamentals of Robotic Surgery (FRS), ensuring that surgeons are equipped with essential skills for modern surgical practice [56–59].

In the era of evidence-based medicine, the outcomes of new technologies and techniques should be closely tracked and analyzed to enable the early identification of harms, allow comparison with existing standards and guide clinical decision-making. This may also strengthen patient trust and informed consent as more accurate and evidence-based information can be provided to patients, enhancing transparency in shared decision-making. To justify the distribution of limited healthcare resources, there is a balance between advocating for individual patients and managing finite resources [60]. While physicians have obligations to both patients and society, professional guidelines emphasize that patient welfare should remain the primary concern [e.g., ACS Code of Professional Conduct, American Board of Internal Medicine (ABIM) Physician Charter]. This fundamental ethical principle should never be compromised by any external pressures such as market forces and administrative demands. The rapid evolution of MIS, exemplified by the early skepticism toward laparoscopy and the eventual adoption, underscores the challenges to justify and balance innovations with patient safety. Nevertheless, the doctrine of Father of Medicine, Hippocrates, Primum non nocere—first do no harm, should be the persistent and lifelong commitment of healthcare professionals in safeguarding patient safety [61].

#### Blurring the lines: medicolegal issues and legal challenges in MIS

The legal landscape surrounding MIS is shaped by a combination of country-specific legal doctrines and widely accepted medical ethics principles. While some medicolegal standards—such as informed consent, duty of care, and the expectation of surgeon competency—are broadly applicable across jurisdictions, others are rooted in specific legal precedents. Notably, the Bolam test (UK, 1957), Bolitho refinement (UK, 1997), Montgomery ruling (UK, 2015), and the Modified Montgomery test (Singapore, 2017) form the basis of common law interpretations of surgical negligence. These are contrasted with evolving frameworks in civil law jurisdictions, where codified standards and regulatory oversight may take precedence.

The term “medicolegal” pertains to the intersection of medical concerns and legal principles. This broadly encompasses cases related to misdiagnosis, medical malpractice or negligence, patient rights, wrongful injury or death, forensic evaluations, and ethical considerations in healthcare. Many medical vulnerabilities overlap across different fields of medicine, but some are particularly associated with MIS, placing laparoscopic surgeons under intense scrutiny. Understanding medicolegal issues is crucial for healthcare professionals to ensure compliance with legal standards and to uphold ethical medical practices, protecting patient rights and fostering trust in the healthcare system. Negligence was regarded as the most commonly encountered tort for all health professionals [62]. A doctor has certain duties of care toward his patient and any breaches may result in an action for negligence against the doctor [63]. In medical negligence cases, various legal tests have been established to determine and assess the fulfillment of the duty of care. The legal principles governing medical negligence have evolved significantly over time, shaping the standard of care expected from medical practitioners. The Bolam test (1957) established that a doctor was not liable for negligence if their actions aligned with a practice accepted as proper by a responsible body of medical professionals skilled in the relevant field, even if another body of opinion held a different view [64]. This principle was later refined by the Bolitho addendum (1997), which introduced an additional requirement that the professional opinion relied upon must satisfy a threshold test of logic. If the opinion failed to withstand logical analysis, the court had the authority to disregard it [65].

A further shift occurred with the Montgomery test (2015), which moved the focus from medical professionals’ standards to patient autonomy and informed consent [66]. Under this test, a doctor was obligated to disclose risks or alternative treatments that a reasonable patient in a similar position would find material, as well as those that the specific patient would have considered significant based on information known or reasonably expected to be known by the doctor. However, exceptions to disclosure existed in cases where it would be seriously detrimental to the patient’s health or when necessity justified withholding information. The Modified Montgomery test (2021), formulated by the Singaporean Court of Appeal, further refined the principles of disclosure by introducing a structured three-stage approach: [1] sufficiency of information [2], possession of information, and [3] justification for withholding information [67]. This framework provided a systematic method for assessing a physician’s duty to disclose, reinforcing the emphasis on patient-centered care while maintaining legal safeguards for medical professionals.

There was a rapid increase trend in the medicolegal liability claims arising from MIS [63]. This might be secondary to the wide adoption of MIS into the practice, accompanied by an increase in the incidence of associated iatrogenic complications [68]. A substantial number of these complications were related to electrosurgical procedures, arising from surgical pilot error, improper usage or maintenance of electrosurgical instruments, or burns occurring outside the surgeon’s direct line of sight or control. The increasing number of patient injury reports, insurance claims, and legal cases has highlighted the risks associated with intra-abdominal electrosurgical injuries during laparoscopic procedures. These have triggered the Association of Trial Lawyers of America (ATLA) to establish a special Laparoscopic Litigation Group in 1994. In response to the emerging medicolegal concerns, the Consortium on Electrosurgical Safety During Laparoscopy had gathered experts to examine strategies in 1997 [68]. The increased litigation risk associated with specific surgical errors had led malpractice insurers to raise premiums for surgeons performing these procedures by 15–20%. Ultimately, the financial burden would extend to both hospitals and patients [69].

It is noteworthy that legal professionals have increasingly become prominent advocates in addressing complications arising from MIS, often shaping discourse on patient safety and surgical accountability. There are an increasing number of law firms now specialized in legal services related to MIS, reflecting the high volume of claims associated with surgical complications in this field [70–72]. From a patient safety perspective, this provides legal resources for victims of surgical errors, promoting accountability and higher standard of care. However, for surgeons and hospitals, this may eventually contribute to defensive medicine, further increase malpractice insurance costs and healthcare expenses. Ideally, the improvement of surgical training, standardization of protocols, and enhancement of patient safety measures should be the focuses in minimizing complications and reducing the need for legal claims.

#### Medicolegal vulnerabilities in MIS: insights from gynecology with broader surgical implications

While this review draws examples from gynecological surgery due to its leading role in pioneering MIS, many of the medicolegal vulnerabilities described—such as access-related injuries, thermal damage, and incomplete consent—are common across all laparoscopic and robotic surgical disciplines, including general, colorectal, urologic, and thoracic surgery.

Gynecologists are pioneers in MIS, particularly laparoscopic approaches, starting in the 1960s [73]. Currently, MIS is the most popular method of surgical intervention in gynecology which includes hysteroscopy, cystoscopy, vaginal surgery, and laparoscopy [74]. In addition, MIS is regarded as an essential skill for all gynecologists [75]. Similar to MIS in other fields of surgery, the implementation of MIS in gynecology challenged gynecologists with new legal and ethical issues [76]. Several key factors contribute to medicolegal vulnerabilities, ranging from preoperative considerations to intraoperative decisions and post-operative management. Critical areas of concern include diagnosis accuracy, surgical choice, informed consent, surgeon expertise, patient selection, complication disclosure, and physiological adaptation [77]. Additionally, issues related to thermal injury, facility standards, and thorough documentation play a significant role in mitigating legal risks and ensuring patient safety [78].

Misdiagnosis and delayed diagnosis are significant concerns in minimally invasive surgery, particularly in diagnostic laparoscopy. While laparoscopy offers superior visual inspection compared to imaging in evaluating abdominal or pelvic pain, staging cancers and assessing injuries, it carries inherent risks and challenges [79]. Preoperative misdiagnosis may lead to unnecessary surgical intervention and expose patients to avoidable risks. Intraoperative misdiagnosis, often caused by a limited visual field or technical challenges, may result in undetected conditions such as malignancies [80]. These diagnostic errors highlight the need for meticulous preoperative assessment and intraoperative vigilance to reduce potential legal complications.

The selection of appropriate surgical indications and techniques is paramount in mitigating medicolegal risks. The use of laparoscopy in patients with severe adhesions, where an open approach may be safer, poses a risk of intraoperative complications and subsequent litigation. Conversely, failure to offer MIS when it is the established standard of care may be regarded as a deviation from accepted clinical practice [75, 79]. For example, hysteroscopy is the preferred modality for myomectomy in submucosal fibroids, necessitating preoperative ultrasound assessment to determine its feasibility based on factors, such as myoma size, location, and proximity to the serosal surface [81]. Similarly, procedures like laparoscopic colorectal resections also require detailed preoperative mapping, particularly in cases involving complex adhesions or tumors near critical structures. In gynecologic oncology, laparoscopic pelvic lymphadenectomy and radical vaginal hysterectomy have emerged as recognized alternatives to traditional open procedures for early stage cervical cancer. Therefore, adherence to evidence-based surgical guidelines and thorough preoperative evaluation are essential in minimizing litigation risks while optimizing patient outcomes [79].

Informed consent is a fundamental component of MIS. Comprehensive disclosure should include the surgeon’s level of experience with MIS, potential procedural risks (such as equipment failure, conversion to open surgery, and injury to adjacent organs), and alternative treatment options, including non-surgical approaches [82]. Patients must also be informed of the consequences and risks associated with rejecting the proposed treatment or procedure [83]. Incomplete disclosure of potential complications in MIS results in significant medicolegal risks. Adverse reactions to local anesthetics during laparoscopy may lead to allegations of overtreatment, while adjacent organ injuries such as uterine perforation occurring during cervical dilation for severe stenosis can cause claims of negligence [84, 85]. Ensuring comprehensive preoperative counseling and obtaining informed consent are critical in mitigating these risks. Additionally, the prophylactic use of antibiotics may serve as a protective measure against negligence claims in cases of post-operative pyrexia of unknown origin [85]. Complex complications, such as intestinal and uterus perforation, are often underestimated intraoperatively, with delayed onset of peritonitis post-discharge posing further medicolegal implications. Furthermore, the decision to convert to open surgery must be meticulously documented and explicitly justified as being in the patient’s best interest. Other potential complications necessitating thorough preoperative discussion include the physiological effects of abdominal insufflation and the Trendelenburg position on organ function, as well as thermal injuries to adjacent tissues resulting from electrosurgical techniques [75, 86, 87]. Effective risk communication, comprehensive documentation, and adherence to established surgical safety protocols are essential in minimizing medicolegal liability in MIS.

Additionally, managing patient expectations is essential particularly regarding recovery time, scarring, pain, and potential complications, to prevent misconceptions and dissatisfaction [88–91]. Offering a second opinion, particularly for procedures like hysteroscopy, further reinforces ethical and legal standards in patient care. Essential information that must be conveyed includes the purpose, utility, and method, and duration of the procedure. Patients should also be informed about the necessity of medications, specifying their type, route of administration, dosage, side effects, and potential interactions with concurrent treatments.

Patient selection is a critical determinant of outcomes in minimally invasive surgery (MIS), as a one-size-fits-all approach may lead to suboptimal results [75]. Several patient-related factors, including body mass index (BMI), prior surgical history, comorbidities, and complex anatomical variations, must be considered when determining surgical eligibility. Comorbid conditions significantly impact perioperative risk. In laparoscopic procedures, abdominal insufflation elevates intra-abdominal pressure, shifting the diaphragm cephalad and leading to reduced lung compliance, decreased functional residual capacity, and increased airway pressures with V/Q mismatch [75]. Trendelenburg positioning further exacerbates these respiratory effects, necessitating careful intraoperative monitoring. Obesity presents additional challenges across all surgical stages. Preoperatively, it increases the risk of comorbidities. Intraoperatively, excessive adipose tissue and a thickened abdominal wall impair visualization, elevate baseline intra-abdominal pressure, and increase the risk of incorrect Veress needle entry [75]. Postoperatively, enhanced monitoring is required for pulmonary, thromboembolic, and glycemic complications.

A surgeon’s proficiency in MIS depends on formal training, experience, technical skill, and the ability to manage complications, including timely conversion to open surgery when necessary. Competency is particularly critical in MIS due to its technical complexity, limited direct access, and high patient expectations [92]. To ensure proficiency, credentialing requires completion of structured training programs, certification, proctoring, and mentorship [76]. Ongoing professional development through Continuing Medical Education (CME) and simulation training is essential for maintaining competence. Inadequate training and failure to stay updated on evolving techniques compromise surgical outcomes. Deficiencies in perioperative care, such as insufficient patient education on warning signs and inadequate follow-up planning, may lead to preventable complications [82]. For instance, hysteroscopic procedures require careful monitoring of fluid deficits and adherence to established management protocols to prevent adverse outcomes. Healthcare institutions play a critical role in ensuring the competence of surgeons and their assisting personnel, including residents, through adequate supervision and training. Proper use of technology and surgical instruments is essential for maintaining procedural safety and efficiency. Additionally, hospitals must ensure the availability, proper maintenance, and functionality of necessary medical devices [82]. Institutional responsibility also extends to maintaining comprehensive and accurate medical records to support quality assurance and patient safety.

The discrepancies between histopathological examination (HPE) findings and preoperative diagnoses in minimally invasive gynecological procedures lead patients to question the appropriateness of surgical decisions, especially when there is a delay in treatment for the actual pathology [93]. For instance, a presumed benign cyst excised laparoscopically may later be identified as malignant, postponing essential cancer staging and treatment. Similarly, lymph nodes removed due to suspected lymphoma may ultimately show only reactive hyperplasia, raising concerns about the necessity of the procedure and causing patient distress. Several intraoperative factors further complicate MIS, including limited visualization, which may hinder the detection of subtle abnormalities. Additionally, electrosurgical burns often remain undetected during surgery, as they occur outside the surgeon’s keyhole field of view [94]. Sampling errors, such as inadequate biopsy specimens, may further compromise diagnostic accuracy. Histopathological factors, such as errors in specimen handling and labeling, can affect diagnostic reliability, while electrocautery-induced tissue charring may distort surgical margins, potentially impacting cancer staging. Furthermore, pathological variability poses an additional challenge, influencing the consistency and accuracy of histological assessments [95]. In conclusion, the medicolegal vulnerabilities associated with minimally invasive surgery (MIS) underscore the need for meticulous surgical planning, intraoperative vigilance, and robust post-procedural protocols.

Medicolegal standards in MIS vary substantially between countries, shaped by distinct legal traditions and healthcare structures. In common law systems like the United Kingdom, Malaysia, and the United States, case law plays a defining role—exemplified by the Bolam (1957), Bolitho (1997), and Montgomery (2015) tests that guide surgical liability and informed consent. Conversely, in civil law jurisdictions, such as Germany, France, and Japan, codified statutes and administrative regulations are more prominent, with less reliance on judicial precedent. In the United States, tort law varies by state and often results in higher litigation costs, encouraging defensive medicine. In contrast, some European systems rely on no-fault compensation models, reducing blame but emphasizing transparency and reporting [96].

These global differences have significant implications for how MIS is practiced and taught. Surgeons operating in international or multicultural contexts must be aware of local expectations for documentation, consent, and professional accountability. As MIS technologies evolve—particularly with AI integration and robotic platforms—there is a growing need for cross-border dialog to harmonize safety standards and address emerging legal challenges.

## Conclusion

Minimally invasive surgery (MIS) has transformed surgical practice, offering enhanced patient outcomes while evolving from ancient techniques to advanced robotic-assisted procedures. However, its complexity introduces medicolegal challenges, including informed consent, patient selection, surgeon competency, institutional responsibility, and diagnostic discrepancies. Addressing these issues requires rigorous training, credentialing, and adherence to legal and ethical standards. Future advancements, particularly in artificial intelligence and robotics, promise greater precision, task automation, and objective proficiency assessments. However, as technology progresses, legal frameworks must adapt to balance innovation with patient safety.

## Data Availability

No datasets were generated or analyzed during the current study.

## References

[1] Ochsner JL (2000) Minimally invasive surgical procedures. Ochsner J 2(3):135–136 21765681 PMC3117518

[2] Zhao Z, Gu J (2022) Open surgery in the era of minimally invasive surgery. Chin J Cancer Res 34(1):63–65 35355929 10.21147/j.issn.1000-9604.2022.01.06 PMC8913255

[3] Siddaiah-Subramanya M, Tiang KW, Nyandowe M (2017) A new era of minimally invasive surgery: progress and development of major technical innovations in general surgery over the last decade. Surg J (N Y) 3(4):e163–e166 29134202 10.1055/s-0037-1608651 PMC5680046

[4] Hargest R (2020) Five thousand years of minimal access surgery: 3000BC to 1850: early instruments for viewing body cavities. J R Soc Med 113(12):491–496 33135953 10.1177/0141076820967913 PMC7816654

[5] Mattelaer JJ, Billiet I (1995) Catheters and sounds: the history of bladder catheterisation. Paraplegia 33(8):429–433 7478735 10.1038/sc.1995.95

[6] Driver G, Miles J (1955) Codex Hammurabi. Babylonian Laws. Clarendon Press, Oxford, p 2

[7] Singhal G, Singh L (1981) Ancient Indian surgery based on Susrata Samhita, vol I–X. Singhal Publications, Varanasi (Banaras)

[8] Singh V (2017) Sushruta: the father of surgery. Natl J Maxillofac Surg 8(1):1–3 28761269 10.4103/njms.NJMS_33_17 PMC5512402

[9] Rudiman R (2021) Advances in gastrointestinal surgical endoscopy. Ann Med Surg 72:103041 10.1016/j.amsu.2021.103041 PMC8636781 34888040

[10] Schollmeyer T, Mettler L, Ruther D, Alkatout I (2013) Practical manual for laparoscopic & hysteroscopic gynecological surgery. JP Medical Ltd, London

[11] Amr SS, Tbakhi A (2007) Abu al Qasim al Zahrawi (Albucasis): pioneer of modern surgery. Ann Saudi Med 27(3):220–221 17575478 10.5144/0256-4947.2007.220 PMC6077085

[12] Gurunluoglu R, Shafighi M, Gurunluoglu A, Cavdar S (2011) Giulio Cesare Aranzio (Arantius)(1530–89) in the pageant of anatomy and surgery. J Med Biogr 19(2):63–69 21558532 10.1258/jmb.2010.010049

[13] de Ronsil GA, Hunter W. Memoires de chirurgie, avec quelques remarques historiques sur l’etat de la médecine & de la chirurgie en France & en Angleterre. Par George Arnaud, docteur en médecine, ancien membre de l’académie royale de chirurgie de Paris, & un des professeurs en l’ecole de St. Cosme, membre de la société des chirurgiens de Londres. Premiere [-seconde] partie: chez J. Nourse, libraire du Roi, dans le Strand.; 1768.

[14] Herr H (2009) Civiale, stones and statistics: The dawn of evidence-based medicine. BJU Int 104:300–302 19466952 10.1111/j.1464-410X.2009.08529.x

[15] Sircus W (2003) Milestones in the evolution of endoscopy: a short history. J R College Phys Edin 33(2):124–134 12833908

[16] Desormeaux AJ (1865) De l’endoscope et de ses applications au diagnostic et au traitement des affections de l’urethre et de la vessie. Baillière

[17] Reuter MA, Reuter HJ, Engel R (1999) History of endoscopy Vol. I-IV. MaxNitzeMuseum, Stuttgart, Germany 1:8

[18] Hatzinger M, Fesenko A, Sohn M (2014) The first human laparoscopy and NOTES operation: Dimitrij Oscarovic Ott (1855–1929). Urol Int 92(4):387–391 24852454 10.1159/000358016

[19] Litynski GS (1997) Laparoscopy-the early attempts: spotlighting Georg Kelling and Hans Christian Jacobaeus. J Soc Laparoendosc Surg 1(1):83 PMC3015224 9876654

[20] Hargest R (2020) Five thousand years of minimal access surgery: 1850 to 1990: technological developments. J R Soc Med 114(1):19–29 33135950 10.1177/0141076820967918 PMC8173353

[21] Veress J (1938) Neues instrument zur ausführung von brust-oder bauchpunktionen und pneumothoraxbehandlung. DMW-Deutsche Medizinische Wochenschrift 64(41):1480–1481

[22] Orndoff B (1920) The peritoneoscope in diagnosis of diseases of the abdomen. J Radiol 1:307

[23] Kalk H (1929) Erfahrungen mit der Laparoskopie.(Zugleich mit Beschreibung eines neuen Instrumentes.) Z. klin. Med.

[24] Hasson H (1971) A modified instrument and method for laparoscopy. Am J Obstet Gynecol 110:886–887 4254516 10.1016/0002-9378(71)90593-x

[25] Klein R, Palmer R (1961) Technique of sampling human ova by follicular puncture under celioscopy. C R Seances Soc Biol Fil 155:1919–1921 14456774

[26] Semm K (1983) Endoscopic appendectomy. Endoscopy 15(02):59–64 6221925 10.1055/s-2007-1021466

[27] Bhatt J, Jones A, Foley S, Shah Z, Malone P, Fawcett D et al (2010) Harold Horace Hopkins: a short biography. BJU Int 106(10):1425–1428 21049584 10.1111/j.1464-410x.2010.09717.x

[28] Whitaker M (2012) Saints and Sinners Harold Hopkins. Bull R College Surg Eng 94(5):168–170

[29] Hirschowitz B (1963) A fibre optic flexible oesophagoscope. The Lancet 282(7304):388 10.1016/s0140-6736(63)93065-4 14044296

[30] Ruddock JC (1949) The application and evaluation of peritoneoscopy. Calif Med 71(2):110 18134893 PMC1520099

[31] SLS MEMBERS DO AMAZING THINGS: Society of Laparoscopic & Robotic Surgeons; https://sls.org/nezhats-history-of-endoscopy/chapter-16/.

[32] Hargest R (2020) Five thousand years of minimal access surgery: 1990–present: organisational issues and the rise of the robots. J R Soc Med 114(2):69–76 33135951 10.1177/0141076820967907 PMC7879007

[33] Morgenstern L (2006) George Berci: past, present, and future. Surg Endosc 20(Suppl 2):S410–S411 16544065 10.1007/s00464-006-0030-7

[34] Du Y, Long Q, Guan B, Mu L, Tian J, Jiang Y et al (2018) Robot-assisted radical prostatectomy is more beneficial for prostate cancer patients: a system review and meta-analysis. Med Sci Monit 24:272–287 29332100 10.12659/MSM.907092 PMC5776881

[35] National Bowel Cancer Audit Annual Report 2018. 2018.

[36] Greaves N, Nicholson J (2011) Single incision laparoscopic surgery in general surgery: a review. Ann R Coll Surg Engl 93(6):437–440 21929912 10.1308/003588411X590358 PMC3369327

[37] Kavic MS, Mirza B, Horne W, Moskowitz JB (2008) NOTES: issues and technical details with introduction of NOTES into a small general surgery residency program. JSLS 12(1):37–45 18402737 PMC3016031

[38] Hamilton A (2024) The future of artificial intelligence in surgery. Cureus 16(7):e63699 39092371 10.7759/cureus.63699 PMC11293880

[39] Zemmar A, Lozano AM, Nelson BJ (2020) The rise of robots in surgical environments during COVID-19. Nat Mach Intell 2(10):566–572

[40] Morris MX, Fiocco D, Caneva T, Yiapanis P, Orgill DP (2024) Current and future applications of artificial intelligence in surgery: implications for clinical practice and research. Front Surg. 10.3389/fsurg.2024.1393898 38783862 10.3389/fsurg.2024.1393898 PMC11111929

[41] Iftikhar M, Saqib M, Zareen M, Mumtaz H (2024) Artificial intelligence: revolutionizing robotic surgery: review. Ann Med Surg. 10.1097/MS9.0000000000002426 10.1097/MS9.0000000000002426 PMC11374272 39238994

[42] Mandler AG (2018) Touch surgery: a twenty-first century platform for surgical training. J Digit Imaging 31(5):585–590 29956010 10.1007/s10278-018-0102-y PMC6148812

[43] Riddle EW, Kewalramani D, Narayan M, Jones DB (2024) Surgical simulation: virtual reality to artificial intelligence. Curr Probl Surg 61(11):101625 39477664 10.1016/j.cpsurg.2024.101625

[44] St John A, Caturegli I, Kubicki NS, Kavic SM (2020) The rise of minimally invasive surgery: 16 year analysis of the progressive replacement of open surgery with laparoscopy. JSLS. 10.4293/JSLS.2020.00076 33510568 10.4293/JSLS.2020.00076 PMC7810432

[45] Bingmer K, Ofshteyn A, Stein SL, Marks JM, Steinhagen E (2020) Decline of open surgical experience for general surgery residents. Surg Endosc 34(2):967–972 31183795 10.1007/s00464-019-06881-0

[46] Starnes BW (2013) A surgeon’s perspective regarding the regulatory, compliance, and legal issues involved with physician-modified devices. J Vasc Surg 57(3):829–831 23446124 10.1016/j.jvs.2012.11.043

[47] Strong VE, Forde KA, MacFadyen BV, Mellinger JD, Crookes PF, Sillin LF et al (2014) Ethical considerations regarding the implementation of new technologies and techniques in surgery. Surg Endosc 28:2272–2276 24962863 10.1007/s00464-014-3644-1

[48] Sachdeva AK, Russell TR (2007) Safe introduction of new procedures and emerging technologies in surgery: education, credentialing, and privileging. Surg Clin North Am 87(4):853–866 17888784 10.1016/j.suc.2007.06.006

[49] SAGES Resource Guide. https://www.sages.org/about/resources/.

[50] Pugh J (2020) Autonomy, rationality, and contemporary bioethics. Oxford University Press, Oxford 32396289

[51] Weaver JP (1984) The problem with the operative permit. Surg Gynecol Obstet 159(6):579–580 6390760

[52] Weaver JP (1987) Beyond the operative permit. N C Med J 48(2):74 3470608

[53] Wilson MR (2003) The new ACGME resident duty hours: big changes, bigger challenges. Ochsner J 5(2):3–5 22826676 PMC3399327

[54] Fitzgibbons SC, Chen J, Jagsi R, Weinstein D (2012) Long-term follow-up on the educational impact of ACGME duty hour limits: a pre-post survey study. Ann Surg 256(6):1108–1112 23069864 10.1097/SLA.0b013e31825ffb33

[55] Carney CM, Stampler KM, McKoy L (2021) Implementing FLS Training Protocol for Minimally Invasive GYN Surgery Training. J Minim Invasive Gynecol 28(11):S112–S113

[56] SAGES/MIRA Consensus Document on Robotic Surgery 10.1007/s00464-007-9727-5 18163170

[57] Morgenthal CB, Richards WO, Dunkin BJ, Forde KA, Vitale G, Lin E (2007) The role of the surgeon in the evolution of flexible endoscopy. Surg Endosc 21(6):838–853 17180263 10.1007/s00464-006-9109-4

[58] Hanly EJ, Zand J, Bachman SL, Marohn MR, Talamini MA (2005) Value of the SAGES Learning Center in introducing new technology. Surg Endosc 19(4):477–483 15696360 10.1007/s00464-004-8928-4

[59] Sachdeva AK, Buyske J, Dunnington GL, Sanfey HA, Mellinger JD, Scott DJ et al (2011) A new paradigm for surgical procedural training. Curr Probl Surg 48(12):854–968 22078788 10.1067/j.cpsurg.2011.08.003

[60] Olejarczyk JP, Young M (2024) Patient rights and ethics. StatPearls [Internet]. 30855863

[61] Ravichandran J, Jeganathan R, Woon Teen S (2024) Medical errors, adverse events and patient safety. Recent advances in obstetrics and gynaecology, vol 3. Evangel Publication, New Delhi

[62] Cheluvappa R, Selvendran S (2020) Medical negligence—key cases and application of legislation. Ann Med Surg 57:205–211 10.1016/j.amsu.2020.07.017 PMC7413923 32793340

[63] Pandit MS, Pandit S (2009) Medical negligence: coverage of the profession, duties, ethics, case law, and enlightened defense—a legal perspective. Indian J Urol 25(3):372–378 19881134 10.4103/0970-1591.56206 PMC2779963

[64] Warren JJ (2000) The healthcare professional and the Bolam test. Br Dent J 188(5):237–240 10758684 10.1038/sj.bdj.4800441

[65] Bwana R (2022) Bolam vs. Bolitho: a medico-legal inquiry for a test in medical negligence. Bolitho: a medico-legal inquiry for a test in medical negligence.

[66] Le Gallez I, Skopek J, Liddell K, Kuhn I, Sagar A, Fritz Z (2022) Montgomery’s legal and practical impact: a systematic review at 6 years. J Eval Clin Pract 28(4):690–702 34623013 10.1111/jep.13620

[67] Neo HY (2017) From Bolam-Bolitho to modified-montgomery-a paradigm shift in the legal standard of determining medical negligence in Singapore. Ann Acad Med Singapore 46(9):347–350 29022035

[68] Brill AI, Feste JR, Hamilton TL, Tsarouhas AP, Berglund SR, Petelin JB et al (1998) Patient safety during laparoscopic monopolar electrosurgery–principles and guidelines. Consortium on Electrosurgical Safety During Laparoscopy. JSLS. 2(3):221–225 9876743 PMC3015308

[69] Freeman G (1995) Malpractice insurance goes up for laparoscopic surgeons. Laparosc Surg Updat 3:14–16

[70] Kreisman R (2021) Chicago medical malpractice attorney blog [Internet]. [cited 2025]. Available from: https://www.robertkreisman.com/medical-malpractice-lawyer/2-3-million-settlement-for-negligent-laparoscopic-hysterectomy/.

[71] Firm HL. Texas laparoscopic surgery mistake lawyers. https://www.hastingsfirm.com/texas-surgical-error-attorney/laparoscopic/.

[72] Group AL. Laparoscopic surgery errors [cited 2025]. https://www.arfaalawgroup.com/laparoscopic-surgery-errors.html.

[73] Frumovitz M, Escobar P, Ramirez PT (2011) Minimally invasive surgical approaches for patients with endometrial cancer. Clin Obstet Gynecol 54(2):226–234 21508692 10.1097/GRF.0b013e318218637d PMC5779861

[74] Adamyan LV (2003) Minimally invasive surgery in gynecologic practice. Int J Gynaecol Obstet 82(3):347–355 14499981 10.1016/s0020-7292(03)00216-9

[75] Wesevich V, Webster EM, Baxley SE (2020) Overcoming challenges in minimally invasive gynecologic surgery. Gynecol Pelvic Med 3.

[76] Tanos V, Socolov R, Demetriou P, Kyprianou M, Belle Y, Campo R (2016) Implementation of minimal invasive gynaecological surgery certification will challenge gynaecologists with new legal and ethical issues. Facts Views Vis ObGyn 8:111–118 27909568 PMC5130300

[77] Sandberg EM, Bordewijk EM, Klemann D, Driessen SRC, Twijnstra ARH, Jansen FW (2017) Medical malpractice claims in laparoscopic gynecologic surgery: a Dutch overview of 20 years. Surg Endosc 31(12):5418–5426 28634629 10.1007/s00464-017-5624-8 PMC5715033

[78] Pajuste T, Cerkic HBMSM. Legal perspectives in the modern era of technological transformations.

[79] Roy SN (2006) The risks of laparoscopic surgery: I. Gynecol Surg 3(4):315–319

[80] Fersini F, Maselli V, Miani E, De Palma A, D’Errico A (2021) Misdiagnosis of leiomyosarcomas: case report and medico-legal issues. Gynecol Pelvic Med 4.

[81] Zepiridis LI, Grimbizis GF, Tarlatzis BC (2016) Infertility and uterine fibroids. Best Pract Res Clin Obstet Gynaecol 34:66–73 26856931 10.1016/j.bpobgyn.2015.12.001

[82] Smith SR (1989) Legal issues relating to gynecologic endoscopy. In: Sanfilippo JS, Levine RL (eds) Operative gynecologic endoscopy. New York, Springer, pp 281–298

[83] Eser A, Gümüş İİ, Yüce E, Akgün N, Kalem MN (2015) The management of hysteroscopy complications. Gynecol Obstet Reprod Med 21(2):118–122

[84] Cooper NA, Khan KS, Clark TJ (2010) Local anaesthesia for pain control during outpatient hysteroscopy: systematic review and meta-analysis. BMJ 340:c1130 20332307 10.1136/bmj.c1130 PMC2844502

[85] Thinkhamrop J, Laopaiboon M, Lumbiganon P (2007) Prophylactic antibiotics for transcervical intrauterine procedures. Cochrane Database of Syst Rev. 10.1002/14651858.CD005637 10.1002/14651858.CD005637.pub2 17636811

[86] Nechay TV, Titkova SM, Anurov MV, Mikhalchik EV, Melnikov-Makarchyk KY, Ivanova EA et al (2020) Thermal effects of monopolar electrosurgery detected by real-time infrared thermography: an experimental appendectomy study. BMC Surg 20(1):116 32460827 10.1186/s12893-020-00735-6 PMC7251678

[87] Brinkmann F, Hüttner R, Mehner PJ, Henkel K, Paschew G, Herzog M et al (2022) Temperature profile and residual heat of monopolar laparoscopic and endoscopic dissection instruments. Surg Endosc 36(6):4507–4517 34708296 10.1007/s00464-021-08804-4 PMC9085678

[88] Yamamoto T, Yoshitomi M, Oshimo Y, Nishikawa Y, Hisano K, Nakano K et al (2023) Ability of minimally invasive surgery to decrease incisional surgical site infection occurrence in patients with colorectal cancer and other gastroenterological malignancies. Front Surg. 10.3389/fsurg.2023.1150460 37123540 10.3389/fsurg.2023.1150460 PMC10130529

[89] Gupta AK, Burgos MI, Santiago Rodriguez AJ, Lopez-Viego M, Ramseyer MM (2020) Major bleed post minimally invasive surgical repair of inguinal hernia. Cureus 12(8):e9940 32968600 10.7759/cureus.9940 PMC7505676

[90] Morrow J, Curry D, Dooher M, Woolsey S (2017) Minimally Invasive management of delayed recognition iatrogenic ureteric injury. Ulster Med J 86(3):181–184 29581630 PMC5849975

[91] Marinelli E, Vergallo GM, Tinelli A, Zaami S, Malvasi A (2018) Medicolegal issues on hysteroscopy. In: Tinelli A, Alonso Pacheco L, Haimovich S (eds) Hysteroscopy. Springer International Publishing, Cham, pp 579–590

[92] Chmarra M, Rodrigues S, Jansen F-W, Dankelman J (2011) Challenges in training and assessment of minimally invasive surgical skills. In: Proceedings of the 3rd international conference on computer supported education. pp. 261–268

[93] Kwan JL, Calder LA, Bowman CL, MacIntyre A, Mimeault R, Honey L et al (2024) Characteristics and contributing factors of diagnostic error in surgery: analysis of closed medico-legal cases and complaints in Canada. Can J Surg 67(1):E58–E65 38320779 10.1503/cjs.003523 PMC10852193

[94] Perantinides PG, Tsarouhas AP, Katzman VS (1998) The medicolegal risks of thermal injury during laparoscopic monopolar electrosurgery. J Healthc Risk Manag 18(1):47–55 10176550 10.1002/jhrm.5600180107

[95] Hori Y (2008) Diagnostic laparoscopy guidelines. Surg Endosc 22(5):1353–1383 18389320 10.1007/s00464-008-9759-5

[96] Barbot J, Parizot I, Winance M (2014) “No-fault” compensation for victims of medical injuries. Ten years of implementing the French model. Health Policy 114(2):236–245 24145101 10.1016/j.healthpol.2013.09.004

